# A Neurofeedback-Based Intervention to Reduce Post-Operative Pain in Lung Cancer Patients: Study Protocol for a Randomized Controlled Trial

**DOI:** 10.2196/resprot.4251

**Published:** 2015-05-04

**Authors:** Alessandra Gorini, Chiara Marzorati, Monica Casiraghi, Lorenzo Spaggiari, Gabriella Pravettoni

**Affiliations:** ^1^University of MilanDepartment of Health ScienceMilanItaly; ^2^European Institute of OncologyPsycho-Oncology UnitMilanItaly; ^3^European Institute of OncologyDepartment of Thoracic SurgeryMilanItaly

**Keywords:** acute post-surgical pain, lung cancer, neurofeedback, relaxation, video games, virtual environments

## Abstract

**Background:**

Thoracic surgery appears to be the treatment of choice for many lung cancers. Nevertheless, depending on the type of surgery, the chest area may be painful for several weeks to months after surgery. This painful state has multiple physical and psychological implications, including respiratory failure, inability to clear secretions by coughing, and even anxiety and depression that have negative effects on recovery.

**Objective:**

The aim of this study is to evaluate the effect of a neurofeedback-based intervention on controlling acute post-surgery pain and improving long-term recovery in patients who undergo thoracotomy for lung resection for non-small cell lung cancer (NSCLC) at an academic oncologic hospital.

**Methods:**

This study will be based on a 2-parallel group randomized controlled trial design, intervention versus usual care, with multiple in-hospital assessments and 2 clinical, radiological, and quality of life follow-ups. Participants will be randomized to either the intervention group receiving a neurofeedback-based relaxation training and usual care, or to a control group receiving only usual care. Pain intensity is the primary outcome and will be assessed using the Numeric Pain Rating Scale (NRS) in the days following the operation. Secondary outcomes will include the effect of the intervention on hospital utilization for pain crisis, daily opioid consumption, anxiety, patient engagement, blood test and chest x-ray results, and long-term clinical, radiological, and quality of life evaluations. Outcome measures will be repeatedly taken during hospitalization, while follow-up assessments will coincide with the follow-up visits. Pain intensity will be assessed by mixed model repeated analysis. Effect sizes will be calculated as mean group differences with standard deviations.

**Results:**

We expect to have results for this study before the end of 2016.

**Conclusions:**

The proposed innovative, neurofeedback- and relaxation-based approach to support post-surgery pain management could lead to significant improvements in patient short and long-term outcomes.

##  Introduction

### Background

Lung cancer has been the most common cancer worldwide since 1985, both in terms of incidence and mortality [1-3], and it is among the top five most frequently diagnosed cancers in Italy [4]. Thoracic surgery appears to be the treatment of choice for many lung cancers. Nevertheless, depending on the type of surgery, the chest area may be painful for several weeks to months after surgery. Indeed, after a thoracotomy, patients often suffer from a persistent pain [5-7] due to the skin incision or deeper tissue injuries, costovertebral joint disruption, resection or fracture of ribs or sternum, and further irritation of the pleura by thoracostomy tubes [8,9]. This painful state has multiple implications, including respiratory failure due to limiting inspiration (because deep breathing requires stretching the incision), or an inability to clear secretions by coughing [10]. Acute pain after surgery can become chronic and persist for more than a year in 21%-67% of patients [11,12]. Moreover, a lot of clinical and demographic factors can contribute to the development of chronic postsurgical pain including psychological conditions (anxiety or depression states), previous surgery, other simultaneous pain, injuries of the chest wall, youth, female gender, and increased levels of pain and analgesic use in the perioperative period [13].

Cooley et al [14] have shown that a high level of post-operative pain in lung cancer patients may exacerbate the fear that movement or physical activity will worsen their condition. This belief can lead to catastrophic appraisals of pain sensations that promote a self-perpetuating cycle of behavioral avoidance, hypervigilance, or distress symptoms [15-18], as well as reduced social activity and global perceptions of decreased health [13]. Researchers investigating psychological aspects of persistent pain have shown that the tendency to focus on pain and to negatively evaluate one's ability to deal with pain, pain-related anxiety, fear, and helplessness are associated with increased pain, psychological distress, and physical disability [19]. Post-thoracotomy pain syndrome and its social consequences have been also investigated by a nationwide study in Denmark [20] that highlighted how partial nerve injury and general pain hyperresponsiveness influence daily activities, even 12-36 months after surgery.

These data highlight the importance of finding effective, early interventions in the presence of painful medical procedures [17,19]. Several studies have demonstrated the effectiveness of non-pharmacological techniques (eg, relaxation) that, in addition to traditional treatments, are able to significantly reduce the acute pain and distress associated with invasive medical procedures [21,22]. Patients who undergo relaxation techniques in different health care settings suffering from acute or chronic pain have been shown to experience less pain compared to those who only undergo traditional treatments [23-27]. In particular, Syrjala et al [28] conducted a study to evaluate the effectiveness of cognitive-behavioral techniques and relaxation in reducing cancer-related pain and found that patients who received these type of treatments, in addition to medical care, reported less pain than the control groups. Although further analyses are required [29], relaxation is a non-pharmacological intervention that may control pain in cancer patients [30].

Many non-pharmacological interventions and interactive new technologies, such as video games and virtual reality environments, can greatly impact pain reduction. By playing a game or being immersed in a virtual environment, users experience an attentional competition between a highly salient sensation (pain) and a consciously directed focus on some other information processing activity [31]. The consequence is a reduced pain perception [32-35], as well as observed changes at a neuroanatomical level. Hoffman et al [36,37] conducted an fMRI study to monitor the brain activity in healthy subjects receiving thermal brain stimulation and showed that virtual reality alone significantly reduced the worst pain and pain unpleasantness, as well as pain-related brain activity in the insula and thalamus. Moreover, combined opioid plus virtual reality exposure reduced pain reports more effectively than did opioid alone on all subjective pain measures [38]. These studies demonstrate that, by distracting subjects from a highly salient sensation of pain, virtual reality may change not only the psychological perception of pain, but also the neuroanatomical networks involved in its modulation [39].

Serious games and virtual realities have been used in different contexts to modulate pain perception. In a recent review, Keefe et al [40] affirmed that virtual reality-based behavioral programs can be used to reduce acute or chronic pain among patients undergoing different medical interventions and rehabilitation programs, such as burn wound care, needle-related procedures, intravenous placement, dental treatments, or postoperative pain. In addition, actively participating in distracting tasks have effects not only on concurrent pain experiences, but long term as well, such as the vividness of memories associated with a traumatic event [33], functional performance, energy level, and time of recovery [34].

Since relaxation, distraction, and new technologies have beneficial effects on pain reduction, we propose to implement a research protocol that, by merging these factors, could help post-operative lung cancer patients to cope with acute pain generated by surgery. The technology that best suits our aim is based on the brain-computer interface (BCI) method, which enables a quick measure of brain activity while providing a neurofeedback (based on simple visual or auditory stimuli, or complex virtual environments) to help the user modulate her/his brain activity to accomplish her/his intents [41]. One of the most user-friendly, simple-to-use and low-cost BCI devices on the market is produced by NeuroSky, who sell a non-invasive, dry biosensor that can read electrical activity in the brain to determine attention and relaxation states. The device, called MindWave, is a portable electro encephalogram (EEG) developed to capture neural activity using three dry electrodes (located beneath the ears and the forehead), and decode them by applying specific algorithms. The MindWave device provides information on a user’s delta, theta, alpha, beta, and gamma brainwave band power levels [42]. The power levels can be interpreted by comparing them to themselves, and with each other, to determine relative quantity and temporal fluctuations [43]. Despite that the MindWave device cannot be used to deeply and precisely monitor the EEG brain activity, it is effective in recording the level of attention and relaxation of the user through the analysis of brain wave synchronization and desynchronization [44]. Moreover, the MindWave device works with engaging applications that help users to improve their abilities to reach attentive or relaxed states by giving them specific visual and auditory feedbacks in response to their brain activity.

We believe that the MindWave and its associated applications can benefit patients in the following ways (1) train them in relaxation techniques, (2) engage them in active tasks, and (3) by receiving motivation neurofeedback, push them to continuously improve their performance. Moreover, due to its ease of use, MindWave can be used by patients the precise moments they are experiencing acute pain.

Our goal is to help patients with lung cancer post-operative acute pain gain better control of their symptoms using this innovative, neurofeedback-based pain-control strategy. We hypothesize that patients randomized to receive the intervention will have better pain outcomes, measured by pain intensity, and better medical and psychological outcomes compared with patients receiving usual care.

### Primary Aim

Our primary aim is to evaluate the effect of neurofeedback on pain control in patients with lung cancer who have been recently operated on.

### Secondary Aims

Secondary outcomes include evaluating the effects of neurofeedback on (1) hospital utilization for pain crises, (2) daily opioid consumption, (3) level of anxiety, (4) participants’ pattern of engagement with the MindWave tool, and (5) blood test and chest x-ray results. Long-term outcomes also include clinical, radiological, and quality of life evaluations at the 1 and 4 month follow-ups.

##  Methods

### Trial Design

**Figure 1:**
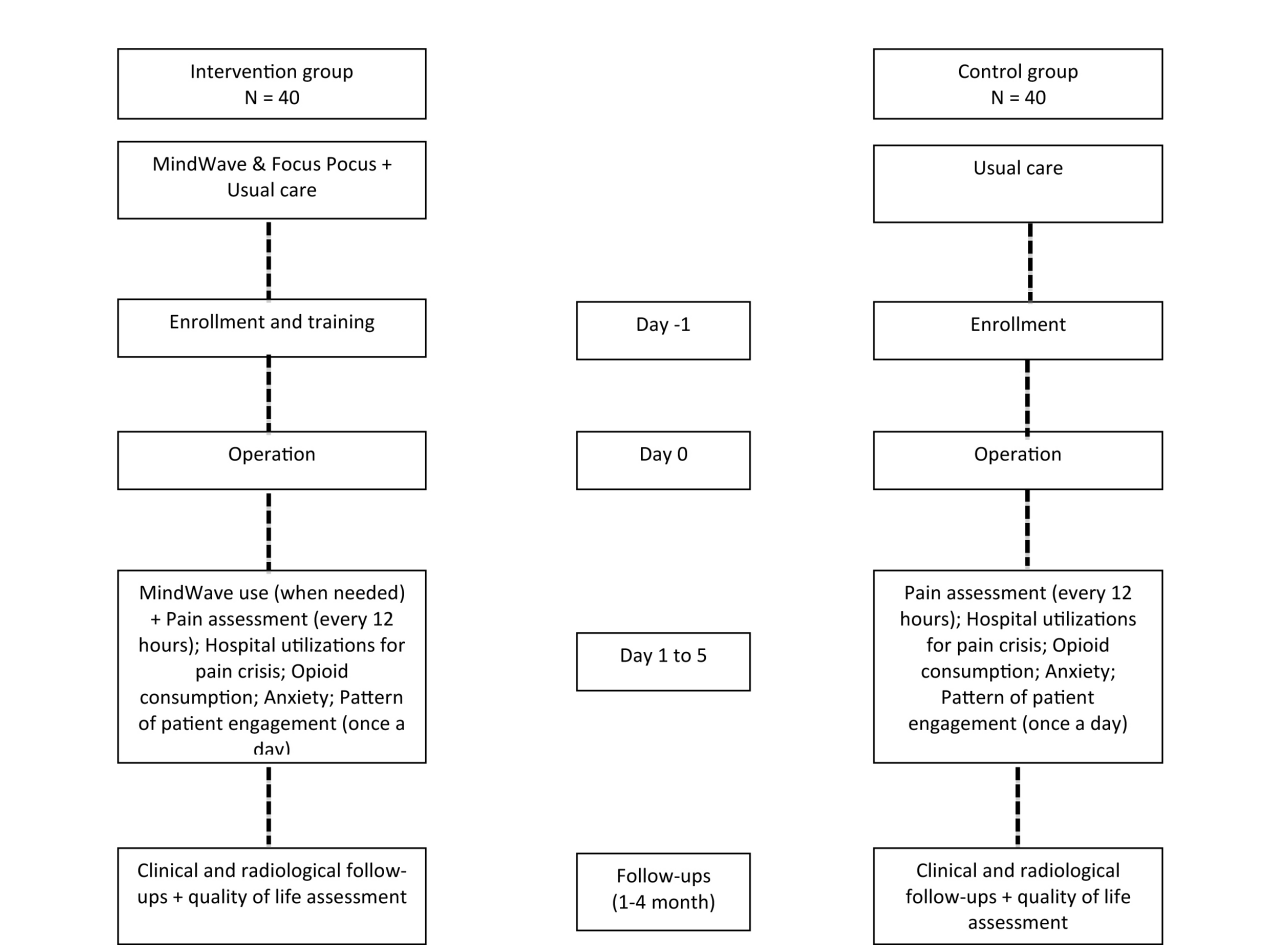
Schematic representation of the trial design.

This study is based on a 2-parallel group randomized controlled trial design (intervention vs usual care), with multiple in-hospital assessments and 2 follow-ups (at 1 and 4 months) based on clinical, radiological, and quality of life assessments. Follow-ups will coincide with the follow-up visits. The research design is shown in Figure 1.

### Participants

Participants included in this study will be recruited from the thoracic surgery unit of the European Institute of Oncology (IEO), Milan, Italy. To be considered eligible to participate, patients must fulfill all of the following inclusion criteria (1) they must be aged ≥18 years, (2) able to consent for self, (3) have a primary diagnosis of non- small cell lung cancer (NSCLC), and (4) they must have undergone a thoracotomy for lung resection <12 hours prior to the first pain assessment. Ineligible patients will be defined as those who (1) have any significant medical or psychiatric comorbidities (other than depression or anxiety), (2) cognitive impediments that would prevent them from being able to utilize the MindWave device, (3) have a known history of substance abuse, and (4) are simultaneously participating in any other research protocols that may have an impact on pain intensity.

### Recruitment Procedure

All patients who are planned to undergo a thoracotomy for lung resection at the European Institute of Oncology, thoracic surgery unit, are considered potential candidates for the present study. Once admitted in the hospital, they will be screened by a trained research assistant to verify their eligibility. Eligible patients will be asked to sign the informed consent, and then randomly assigned to the intervention or control groups. All participants will be instructed to continue to receive medical care from physicians as usual. Subjects included in the intervention arm will be trained on the use of the technology and relaxation technique prior to the operation by a research assistant. They will also receive a MindSet device and tablet with the app Focus Pocus installed on it, for the entire duration of hospitalization (5 days).

### Intervention

#### Framework for Intervention

This neurofeedback-based pain-management program is based on the hypothesis that a relaxation training that provides users an immediate feedback on their performance, as well as a playful environment that moves the patient’s focus from the painful sensation to a specific task can be effective in reducing acute pain perception. In fact, the use of engaging and fun mini-games encourages patients to exercise important psychological processes that underlie their ability to control their own brain responses and, consequently, their behavior.

#### Hardware and Software Equipment

**Figure 2:**
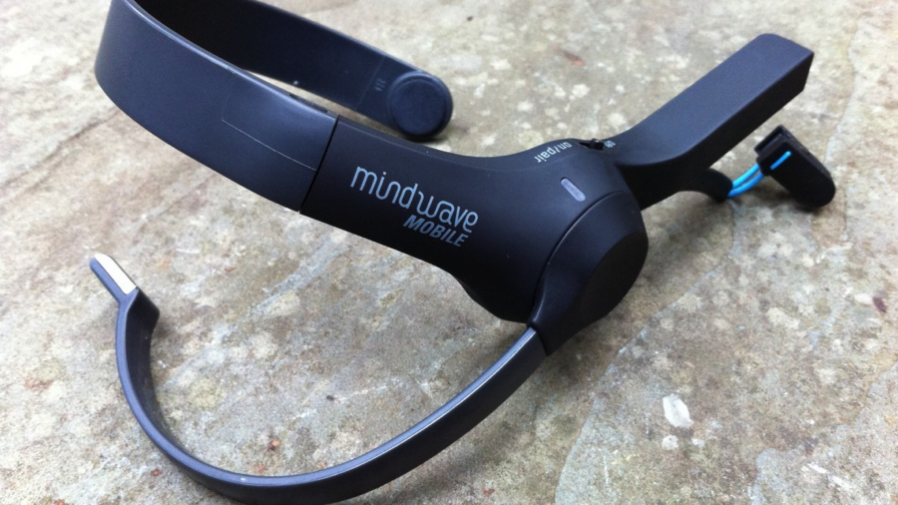
The MindWave mobile device.

Powered by NeuroSky’s Brainwave Technology, the Mindwave headset is a slim, plastic device which fits comfortably, if not unobtrusively, over the user’s left ear (see Figure 2). The Mindwave mobile device uses a single sensor positioned on the forehead to allow users to view their brainwaves in real-time. The Mindwave headset picks up the brain’s electrical activity and divides the signal by frequency into various types of waves, allowing it to infer how relaxed (as measured by alpha and theta waves) or concentrated (as measured by beta and gamma waves) users are. In order to allow the headset to filter out non-brain related electrical activity, a ‘reference’ contact, in the form of a clip that attaches to the earlobe, is included. The MindWave mobile device can connect, via bluetooth, to different devices, and works with most modern operating systems (Windows X or newer, Mac OS X 10.6.5 or newer) and mobile devices running Android or iOS. Its battery life is rated at 8-10 hours with a single AAA battery. Although it will take a minute or two to adjust the headset the first time the user puts it on, setup is relatively simple.

The MindWave mobile costs approximately 100 euros (€) and comes bundled with many free and paid applications, but we limited our choice to those available for the iPad tablet specifically as it is one of the most confortable devices (in terms of portability, weight, and usability) that can be used by bedridden patients. After having tested all the existing iOS-based apps, we opted for the one called “Focus Pocus-BrainControl”. Focus Pocus is a mix of mini-games that uses live brain electrical activity (ie, EEG) from the NeuroSky MindWave device to alter the circumstances of the player. What happens in the game depends on how relaxed the player is. Focus Pocus attracted our attention for the following reasons (1) ease of use, and has a very high-quality interface, yet is low in cost, (2) the games are engaging and fun with a unifying theme, (3) provides cognitive exercises designed by qualified experts, (4) can be used anywhere and anytime without specific supervision, (5) has been already used for scientific purposes [45,46], (6) designed to provide an environment to practice the relaxation skill (other than attention, impulse-control, and memory), (7) can register the user’s training performance in terms of time of use and achievements, and (8) rewards the users’ progress by providing them behavior ratings at the end of each trial. With respect to relaxation, for example, in the Focus Pocus BrainControl games, the outcome of any relaxing experience is the result of the content presented, the environment in which it is presented, and the person’s readiness to learn. This readiness depends on relaxation (a “state” factor) as well as being able to control impulses and ignore internal (into the game) and external (pain sensation) distractions. In order to guarantee improvement in performance, the difficulty levels of the games are adaptive, and can be adjusted on a per game basis to the performance of the users.

**Figure 3:**
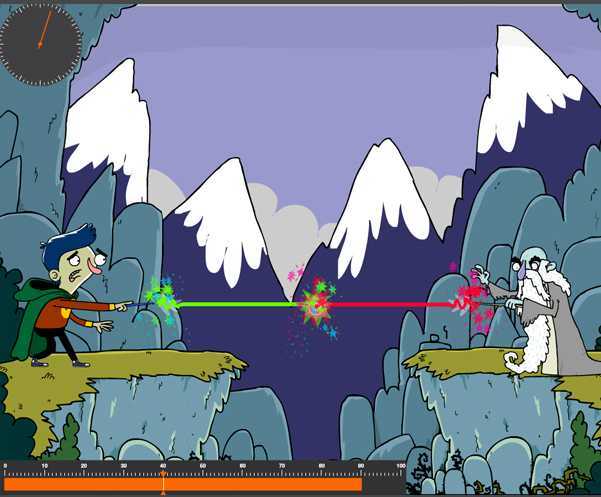
A screenshot of one of the Focus Pocus mini-games.

A screenshot of one of the of the Focus Pocus games is shown in Figure 3. A single electrode on the Neurosky headset (placed on the forehead) is able to pick up a few simple and characteristic brainwaves (created by activity in populations of neurons), some that have been shown to be enriched when the subject is awake and attentive (eg, beta waves), and some when the subject is relaxed (eg, alpha waves). Neurosky has developed algorithms to funnel these and other brain waves into measures of “focus” and “meditation.” In particular, in this game, the player needs to attain a certain level of meditation to win a duel with an evil necromancer. The idea is that through these different activities, the players would be exercising mental capacities that would generalize outside the game (when they experience acute pain, for example).

For the present protocol, 2 provided tablets and MindWave mobile devices will be used at the same time on 2 different patients. They will be given to the patients the day before the operation and left at subjects' bedside for the duration of their hospital stay. The nurses and the patients will be asked to take care of the devices. The MindSet device and its sensor contact points will be cleaned regularly with an alcohol-based cleaner and a soft cloth included in the MindSet casing to prevent cross-infection and to guarantee good signal quality.

### Procedure

#### Training

Prior to the surgery (usually the day before), participants will participate in an in-hospital intensive education (45-60 minutes) training session when they are not yet experiencing post-operative pain. During the training, an expert research assistant will explain to each patient how, when, and for how long the MindWave and the Focus Pocus app should be used. After that, the patient will be encouraged to practice the use of the tool by him/herself under the supervision of the research assistant. Once the patients become autonomous with the device, the training session will end, and the MindWave and tablet to be used until their discharge from the hospital will be provided.

#### Intervention Group (Neurofeedback and Usual Care)

Patients included in the intervention arm will continue to receive the standard care consisting of intercostal analgesia and intravenous pain killers. In addition to it, they will be encouraged to use the MindWave device to manage their pain on-demand and every time they think they need it. During the hospitalization period, patients assigned to the intervention group will also be asked to evaluate their pain over the past 12 hours on a daily basis until their discharge from hospital.

#### Control Group

Subjects assigned to the control arm will receive only the standard care. In adjunct, they will also be asked to evaluate their pain over the past 12 hours, every day starting from the operation until their discharge from hospital.

#### Randomization and Blinding

An independent researcher with no direct contact with the participants will use a computer-generated randomization with a 1:1 ratio and permutated blocks to optimize balance in each treatment arm. Due to the nature of the study, participants, care providers, and researchers cannot be blinded for the allocated treatment. However, the data analysis will be blind, as all of the patients receive a unique study code, under which their data is stored in the database.

### Outcome Evaluation

#### Data Collection Materials

We will assess one primary outcome and several secondary outcomes. A number of validated instruments will be used to assess the outcomes at multiple time points during the hospitalization and follow-ups. The patients, both in the treatment and in the control arms, will be asked to complete them at specific time-points without the supervision of the research assistants. Hospital nurses will be asked if they completed all the outcome measures, as requested by the protocol, at the end of each day. Demographic and clinical information of each participant will be also collected.

#### Primary Outcome

The primary outcome measurement is pain intensity, measured as a continuous outcome. It will be assessed quantitatively with the Numeric Pain Rating Scale (NRS) [47]. The paper version of the questionnaire will be self-administered. Participants will be asked to complete it starting from 12 hours after the operation, and every 12 hours during the entire hospitalization period.

#### Secondary Outcomes

Several secondary outcomes will be measured at various time points during the hospitalization (Textbox 1). Clinical, radiological, and quality of life will be also assessed at the 2 follow-ups (at 1 and 4 months). The clinical and radiological assessment is part of the post-operative routine. However, it will be included in the outcome measures of the present protocol because we expect that a reduction of pain immediately after the operation can result in a better clinical and radiological long-term outcome. Quality of life will be also assessed using the EORTC QLQ-C30 (version 3.0).

**Secondary outcomes measured.**

- The number of events in which the patient reports severe, uncontrolled, and causing distress pain that requires urgent and unplanned care visits
- Opioid consumption, measured quantitatively as oral morphine equivalent daily dose
- Anxiety, measured by the State-Trait Anxiety Inventory (STAI) scale
- The pattern of patient engagement with the MindWave tool, assessed quantitatively by the number and length of time each subject uses it (as recorded by the software). Usability and satisfaction with the tool will be also investigated.
- A blood test and chest x-ray will be also performed at the end of the hospitalization period in order to determine if the intervention group shows a better x-ray outcome and fewer infections, due to more physiotherapy because of reduced pain

### Statistical Analysis

#### Sample Size Estimation

A sample size of 80 subjects, 40 per arm, is sufficient to detect a difference of 1.5 between the two groups (control vs intervention) in pain intensity scores, assuming equal standard deviation of 2.5, using a two-tailed *t* test of difference between means, with 80% power, and a 2-sided alpha of .05. Patients in the experimental group who decide to never use the device during hospitalization will be excluded from the study.

#### Statistical Analysis

Statistical analysis will be conducted with the SPSS Software, version 22, with an alpha of .05 set a priori for all analyses. Descriptive statistics will be used to summarize baseline demographic characteristics by study arm. Continuous variables will be compared between the two groups using a *t* test and categorical variables will be compared using a chi-square test. Pain intensity, our primary outcome, measured longitudinally, will be assessed by mixed model analysis of variance with treatment assignment as the between-group factor and time as the within-subject factor. Effect sizes will be calculated as mean group differences with standard deviations. A similar approach will be used to analyze continuous secondary outcomes while categorical outcomes will be analyzed by chi-square tests.

### Ethics and Informed Consent

The hospital internal ethical committee reviewed and approved the study protocol. Upon meeting eligibility criteria, participants will be informed about the study and asked to sign two copies of the informed consent, one for them and the other for the study team. During the enrollment visit and the entire duration of the study, trained research assistants will be available to answer the patients’ questions and to give them additional information.

## Results

We expect to have results for this study before the end of 2016.

## Discussion

### Principal Findings

The idea to implement this protocol originated from the need to find an on-demand, pain control strategy that helps lung cancer patients to better tolerate acute pain that often arises in the days immediately after surgery. A prompt reduction of pain is fundamental to reduce the risk of respiratory failure and/or the inability to clear secretions by coughing, as well as the probability to develop long-term negative physical and psychological conditions that can significantly interfere with a full recovery.

Biofeedback-based training usually guarantees a persistent learning, even when the machine-guided training ends up [48]. In other words, once users have learned to control their emotions through the machine-guided relaxation training, they usually become able to practice the relaxation techniques without any external help. We argue that this can be applicable to the neurofeedback method, such that, once learned to control pain through relaxation with the help of the MindWave device, patients can continue to use the techniques at home, without the need for any external devices. Therefore, not only effective immediately after the operation, the hospital training can become a great resource to self-manage pain at home. Moreover, giving patients the concrete opportunity to control their pain on-demand is fundamental considering that the most acute pain tends to appear during the night, when the effect of pharmacological treatment decreases and the medical support is at a minimum.

Our main endpoint will serve to evaluate the immediate effect of neurofeedback on pain control. If our results are positive, this technique could be used in combination with traditional pharmacological treatments to improve the patients’ post-operative experience, reduce the use of analgesic, and improve long-term physical and psychological outputs.

A challenge of this research protocol is the use of neurofeedback to reduce pain perception. While relaxation, virtual reality, and gaming have been demonstrated to be effective in reducing pain, there is no data on the efficacy of neurofeedback and related applications. Nevertheless, we believe that neurofeedback has great potential to reduce pain through relaxation for the following three reasons. First, receiving feedback determined by a specific mental activation can facilitate behavioral modifications and learning of relaxation techniques. Second, compared to traditional visual feedback, a more complex feedback coming from a virtual game can encourage a greater involvement of the patient, and consequently, distraction from the painful sensation. Finally the MindWave system is a cheap, user friendly device that can be easily used by patients without the supervision of the research assistant.

### Limitations

The main limitation of the present study is the number of channels and poor precision of the MindWave device in recording the user’s brainwaves. However, since brainwave analyses are not the focus of this study, we do not consider it a critical limitation. Future studies could make use of more sophisticated devices that are currently being advertised but are not yet available on the market.

Another limitation stems from our use of the Focus Pocus app on adult cancer patients, as it was originally developed to train children with specific attentive disorders. Even if the proposed mini-games are suitable for adults, it would be useful, in the future, to develop ad hoc apps tailored to adults’ preferences and abilities.

### Conclusions

To our knowledge, this is the first clinical trial evaluating the impact of a neurofeedback-based intervention on pain management. We hope that our results will lead to larger trials to demonstrate more robust evidence. If our hypotheses are confirmed, the proposed method can be applied to post-operative patients and, in general, to patients suffering from acute pain to reduce care costs and improve overall patient outcomes.

## Abbreviations

BCl: Brain-computer interface
NSCLC: Non-small cell lung cancer
